# Mobile detection of autism through machine learning on home video: A development and prospective validation study

**DOI:** 10.1371/journal.pmed.1002705

**Published:** 2018-11-27

**Authors:** Qandeel Tariq, Jena Daniels, Jessey Nicole Schwartz, Peter Washington, Haik Kalantarian, Dennis Paul Wall

**Affiliations:** 1 Department of Pediatrics, Division of Systems Medicine, Stanford University, California, United States of America; 2 Department of Biomedical Data Science, Stanford University, California, United States of America; Johns Hopkins University, UNITED STATES

## Abstract

**Background:**

The standard approaches to diagnosing autism spectrum disorder (ASD) evaluate between 20 and 100 behaviors and take several hours to complete. This has in part contributed to long wait times for a diagnosis and subsequent delays in access to therapy. We hypothesize that the use of machine learning analysis on home video can speed the diagnosis without compromising accuracy. We have analyzed item-level records from 2 standard diagnostic instruments to construct machine learning classifiers optimized for sparsity, interpretability, and accuracy. In the present study, we prospectively test whether the features from these optimized models can be extracted by blinded nonexpert raters from 3-minute home videos of children with and without ASD to arrive at a rapid and accurate machine learning autism classification.

**Methods and findings:**

We created a mobile web portal for video raters to assess 30 behavioral features (e.g., eye contact, social smile) that are used by 8 independent machine learning models for identifying ASD, each with >94% accuracy in cross-validation testing and subsequent independent validation from previous work. We then collected 116 short home videos of children with autism (mean age = 4 years 10 months, SD = 2 years 3 months) and 46 videos of typically developing children (mean age = 2 years 11 months, SD = 1 year 2 months). Three raters blind to the diagnosis independently measured each of the 30 features from the 8 models, with a median time to completion of 4 minutes. Although several models (consisting of alternating decision trees, support vector machine [SVM], logistic regression (LR), radial kernel, and linear SVM) performed well, a sparse 5-feature LR classifier (LR5) yielded the highest accuracy (area under the curve [AUC]: 92% [95% CI 88%–97%]) across all ages tested. We used a prospectively collected independent validation set of 66 videos (33 ASD and 33 non-ASD) and 3 independent rater measurements to validate the outcome, achieving lower but comparable accuracy (AUC: 89% [95% CI 81%–95%]). Finally, we applied LR to the 162-video-feature matrix to construct an 8-feature model, which achieved 0.93 AUC (95% CI 0.90–0.97) on the held-out test set and 0.86 on the validation set of 66 videos. Validation on children with an existing diagnosis limited the ability to generalize the performance to undiagnosed populations.

**Conclusions:**

These results support the hypothesis that feature tagging of home videos for machine learning classification of autism can yield accurate outcomes in short time frames, using mobile devices. Further work will be needed to confirm that this approach can accelerate autism diagnosis at scale.

## Abstract 2

Dennis P. Wall and colleagues demonstrate that machine learning algorithms can classify children with and without autism spectrum disorder using nonexpert tagging of home videos.

## Author summary

**Why was this study done?:**

- Autism has risen in incidence by approximately 700% since 1996 and now impacts at least 1 in 59 children in the United States.
- The current standard for diagnosis requires a direct clinician-to-child observation and takes hours to administer.
- The sharp rise in incidence of autism, coupled with the un-scalable nature of the standard of care (SOC), has created strain on the healthcare system, and the average age of diagnosis remains around 4.5 years, 2 years past the time when it could be reliably diagnosed.
- Mobile measures that scale could help to alleviate this strain on the healthcare system, reduce waiting times for access to therapy and treatment, and reach underserved populations.

**What did the researchers do and find?:**

- We applied 8 machine learning models to 162 two-minute home videos of children with and without autism diagnosis to test the ability to reliably detect autism on mobile platforms.
- Three nonexpert raters measured 30 behavioral features needed for machine learning classification by the 8 models in approximately 4 minutes.
- Leveraging video ratings, a machine learning model with only 5 features achieved 86% unweighted average recall (UAR) on 162 videos and UAR = 80% on a different and independently evaluated set of 66 videos, with UAR = 83% on children at or under 4.
- The above machine learning process of rendering a mobile video diagnosis quickly created a novel collection of labeled video features and a new video feature–based model with >90% accuracy.

**What do these findings mean?:**

- Short home videos can provide sufficient information to run machine learning classifiers trained to detect children with autism from those with either typical or atypical development. Features needed by machine learning models designed to detect autism can be identified and measured in home videos on mobile devices by nonexperts in timeframes close to the total video length and under 6 minutes.
- The machine learning models provide a quantitative indication of autism risk that provides more granularity than a binary outcome to flag inconclusive cases, potentially adding value for use in clinical settings, e.g., for triage.
- The process of mobile video analysis for autism detection generates a growing matrix of video features that can be used to construct new machine learning models that may have higher accuracy for autism detection in home video.
- Clinical prospective testing in general pediatric settings on populations not yet diagnosed will be needed. However, these results support the possibility that mobile video analysis with machine learning may enable rapid autism detection outside of clinics to reduce waiting periods for access to care and reach underserved populations in regions with limited healthcare infrastructure.

## Introduction

Neuropsychiatric disorders are the single greatest cause of disability due to noncommunicable disease worldwide, accounting for 14% of the global burden of disease [1]. A significant contributor to this metric is autism spectrum disorder (ASD, or autism), which has risen in incidence by approximately 700% since 1996 [2,3] and now impacts 1 in 59 children in the United States [4,5]. ASD is arguably one of the largest pediatric health challenges, as supporting an individual with the condition costs up to $2.4 million during his/her lifespan in the US [6] and over $5 billion annually in US healthcare costs [6].

Like most mental health conditions, autism has a complex array of symptoms [7] that are diagnosed through behavioral exams. The standard of care (SOC) for an autism diagnosis uses behavioral instruments such as the Autism Diagnostic Observation Schedule (ADOS) [8] and the Autism Diagnostic Interview-Revised (ADI-R) [9]. These standard exams are similar to others in developmental pediatrics [10] in that they require a direct clinician-to-child observation and take hours to administer [11–14]. The sharp rise in incidence of autism, coupled with the unscalable nature of the SOC, has created strain on the healthcare system. Wait times for a diagnostic evaluation can reach or exceed 12 months in the US [15], and the average age of diagnosis in the US remains near 5 years of age [2, 13], with underserved populations’ average age at ASD diagnosis as high as 8 years [16–18]. The high variability in availability of diagnostic and therapeutic services is common to most psychiatry and mental health conditions across the US, with severe shortages of mental health services in 77% of US counties [19]. Behavioral interventions for ASD are most impactful when administered by or before 5 years of age [12,20–23]; however, the diagnostic bottleneck that families face severely limits the impact of therapeutic interventions. Scalable measures are necessary to alleviate these bottlenecks, reduce waiting times for access to therapy, and reach underserved populations in need.

As a step toward enabling fast and accurate access to care for ASD, we have used supervised machine learning approaches to identify minimal sets of behaviors that align with clinical diagnoses of ASD [24–30]. We assembled and analyzed item-level outcomes from the administration of the ADOS and ADI-R to train and test the accuracy of a range of classifiers. For the ADOS, we focused our analysis on ordinal outcome data from modules 1, 2, and 3, which assess children with limited or no vocabulary, with phrased speech, and with fluent speech, respectively. Each of the 3 ADOS modules uses approximately 10 activities for a clinical observation of the child at risk and 28–30 additional behavioral measurements used to score the child following the observation. Our machine learning analyses focused on archived records of the categorical and ordinal data generated from the scoring component of these ADOS examinations. Similarly, the ADI-R involves 93 multiple-choice questions asked by a clinician of the child’s primary care provider during an in-clinic interview; as with the ADOS, we focused our classification task on the ordinal outcome data that resulted from the test’s administration.

These preliminary studies focused on building models optimized for accuracy, sparsity, and interpretability that differentiate autism from non-autism while managing class imbalance. We chose models with small numbers of features, with performance at or no more than 1 standard error away from best test performance, and with interpretable outcomes—for example, scores generated by a boosted decision tree or logistic regression (LR) approach. In all, these studies have used score data from 11,298 individuals with autism (mixed with low-, medium-, and high-severity autism) and 1,356 controls (including some children for whom autism may have been suspected but was ruled out) and have identified the following 8 classifiers: a 7-feature alternating decision tree (ADTree7) [29], an 8-feature alternating decision tree (ADTree8) [30], a 12-feature support vector machine (SVM12) [26], a 9-feature LR classifier (LR9) [26], a 5-feature support vector machine (SVM5) [27], a 5-feature LR classifier (LR5) [27], a 10-feature LR classifier (LR10) [27], and a 10-feature support vector machine (SVM10) [27].

Two of these 8 classifiers have been independently tested in 4 separate analyses. In a prospective head-to-head comparison between the clinical outcome and ADTree7 (measured prior to the clinical evaluation and official diagnosis) on 222 children (*N*_ASD_ = 69; *N*_controls_ = 153; median age = 5.8 years), the performance, measured as the unweighted average recall (UAR [31]; the mean of the sensitivity and specificity), was 84.8% [24]. Separately, Bone and colleagues [32] tested the ADTree7 on a “Balanced Independent Dataset” (BID) consisting of ADI-R outcome data from 680 participants (462 ASD, mean age = 9.2 years, SD = 3.1 years) and 218 non-ASD (mean age = 9.4 years, SD = 2.9 years) and found the performance to be similarly high at 80%. Duda and colleagues [25] tested the ADTree8 with 2,333 individuals with autism (mean age = 5.8 years) and 283 “non-autism” control individuals (mean age = 6.4 years) and found the performance to be 90.2%. Bone and colleagues [32] also tested this ADTree8 model in 1,033 participants from the BID—858 autism (mean age = 5.2 years, SD = 3.6 years), 73 autism spectrum (mean age = 3.9 years, SD = 2.4 years), and 102 non-spectrum (mean age = 3.4 years, SD = 2.0 years)—and found the performance to be slightly higher at 94%. These independent validation studies report classifier performance in the range of the published test accuracy and lend additional support to the hypothesis that models using minimal feature sets are reliable and accurate for autism detection.

Others have run similar training and testing experiments to identify top-ranked features from standard instrument data, including Bone [33] and Bussu [34]. These approaches have arrived at similar conclusions, namely that machine learning is an effective way to build objective, quantitative models with few features to distinguish mild-, medium-, and high-severity autism from children outside of the autism spectrum, including those with other developmental disorders. However, the translation of such models into clinical practice requires additional steps that have not yet been adequately addressed. Although some of our earlier work has shown that untrained video annotators can measure autism behaviors on home videos with high interrater reliability and accuracy [35], the question of what steps must be taken to move from minimal behavioral models into clinical practice remains.

The present study builds on this prior work to address this question and the hypothesis that features represented in our minimal viable classifiers can be labeled quickly, accurately, and reliably from short home videos by video raters with no official training in autism diagnosis or child development. We deployed crowdsourcing and real-time video analysis for feature labeling to run and evaluate the accuracy of the 8 machine learning models trained to detect autism in 2 independent home video repositories. This procedure enabled us to test the ability to reduce to practice the process of rapid mobile video analysis as a viable method for identifying autism symptoms and screening. In addition, as the mobile feature tagging of videos automatically generates a rich feature matrix, it presents the opportunity to train a new artificial intelligence model that has potentially higher generalizability to the task of automatic detection of autism in short video clips. We test this related hypothesis by constructing a novel video feature classifier and comparing its results to alternative models in a held-out subset of the original video feature matrix and in an independent external validation set. The results from this work support the hypothesis that autism detection can be done from mobile devices outside of clinical settings with high efficiency and accuracy.

## Methods

### Source classifiers for reduce-to-practice testing

We assembled 8 published machine learning classifiers to test viability for use in the rapid mobile detection of autism in short home videos. For all of the 8 models, the source of training and validation data was medical records generated through the administration of one of two gold-standard instruments in the diagnosis of autism, the ADOS or the ADI-R. The ADOS has several modules containing approximately 30 features that correspond to developmental level of the individual under assessment. Module 1 is used on individuals with limited or no vocabulary. Module 2 is used on individuals who use phrase speech but who are not fluent. Module 3 is used on individuals who are fluent speakers. The ADI-R is a parent-directed interview that includes >90 elements each asked of the parent, with multiple choices for answers. Each model was trained on item-level outcomes from the administration of either the ADOS and ADI-R and optimized for accuracy, sparsity of features, and interpretability.

For the purpose of brevity without omission of detail, we opted to create an abbreviation for each model using a basic naming convention. This abbreviation took the form of “model_type”-“number of features.” For example, we used ADTree8 to refer to the use of an alternating decision tree (ADTree) with 8 features developed from medical data from the administration of the diagnostic instrument ADOS Module 1, and LR5 to refer to the LR with 5 behavioral features developed from analysis of ADOS Module 2 medical record data, and so on.

#### ADTree7

We (Wall and colleagues [29]) applied machine learning to electronic medical record data recorded through the administration of the ADI-R in the diagnostic assessment of children at risk for autism. We used an 80%:20% training and testing split and performed 10-fold cross-validation for a sample of 891 children with autism and 75 non-autism control participants with an ADTree model containing 7 features. The ADTree uses boosting to manage class imbalance [36,37]. We also performed up-sampling through 1,000 bootstrap permutations to manage class imbalance. The model was validated in a clinical trial on 222 participants [24] and in a BID consisting of 680 individuals (462 with autism) [32]. The lowest sensitivity and specificity exhibited were 89.9 and 79.7, respectively (UAR = 84.8%).

#### ADTree8

We [30] used a dataset of score sheets from ADOS Module 2 for 612 children with ASD and 15 non-autism control participants with a 90%:10% training and testing split and 10-fold cross-validation to train and test an ADTree model with 8 of the 29 Module 2 features. The ADTree uses boosting and has inherent robustness to class imbalance [36,37]. We also performed up-sampling through 1,000 bootstrap permutations to test the sensitivity of model performance to class imbalance. This 8-feature ADTree model was independently tested on 446 individuals with autism by Wall and colleagues [30], on 2,333 individuals with autism and 238 without autism by Duda and colleagues [25], and on 1,033 individuals (858 autism, 73 autism spectrum, 102 non-spectrum) by Bone and colleagues [32]. The lowest sensitivity and specificity reported were 97.1% and 83.3%, respectively (UAR = 90.2%).

#### LR9

We [26] performed training with ADOS Module 2 records on 362 individuals with autism and 282 individuals without autism with backward feature selection and iterative removal of the single lowest-ranked feature across 10 folds each with a 90%:10% class split. Classes were weighted inversely proportional to class size to manage imbalance. The model with the highest sensitivity and specificity and lowest number of features, LR with L1 regularization and 9 features, was selected for testing. We tested the model on independent data from 1,089 individuals with autism and 66 individuals with no autism diagnosis. The lowest sensitivity and specificity identified were 98.8% and 89.4%, respectively (UAR = 94.1%).

#### SVM12

We [26] used score sheets from ADOS Module 3 generated by the evaluation of 510 children with ASD and 93 non-ASD control participants. These data were split into a 90% training and 10% testing set. Training and parameter tuning were performed with stepwise backward feature selection and iterative removal of the single lowest-ranked feature across 10 folds. Classes were weighted inversely proportional to class size to manage imbalance. Several models were fit to each of the feature cross-validation folds. The model with the highest sensitivity and specificity and lowest number of features, a Support Vector Machine (SVM) with a radial basis function, was then applied to the test set to measure generalization error. We tested the model on 1,924 individuals with autism and 214 individuals who did not qualify for an autism diagnosis. The lowest sensitivity and specificity identified on the test set were 97.7% and 97.2%, respectively (UAR = 97.5%).

#### LR5 and SVM5

In this experiment, we [27] used medical records generated through the administration of ADOS Module 2 for 1,319 children with autism and 70 non-autism control participants. The dataset was split 80%:20% into train and test sets, with the same proportion for participants with and without ASD in each set. Class imbalance was managed by setting class weights inversely proportional to the class sizes. A 10-fold cross-validation was used to select features, and a separate 10-fold cross-validation was run for hyperparameter tuning prior to testing the performance. An SVM and an LR model with L1 regularization showed the highest test performance with 5 features. The lowest sensitivity and specificity exhibited on the test set for SVM5 were 98% and 58%, respectively, (UAR = 78%) and 93% and 67%, respectively, (UAR = 80%) for LR5.

#### LR10 and SVM10

In this experiment, we [27] used medical records generated through the administration of ADOS Module 3 for 2,870 children with autism and 273 non-autism control participants. The dataset was split 80%:20% into train and test sets, with the same proportion for participants with and without ASD in each set. Class imbalance was managed by setting class weights inversely proportional to the class sizes. A 10-fold cross-validation was used to select features, and a separate 10-fold cross validation was run for hyperparameter tuning prior to testing the performance. An SVM and an LR model with L1 regularization showed the highest test performance with 10 features. The lowest sensitivity and specificity exhibited on the independent test set for SVM10 were 95% and 87%, respectively, (UAR = 91%) and 90% and 89%, respectively, (UAR = 89.5%) for LR10.

**Fig 1:**
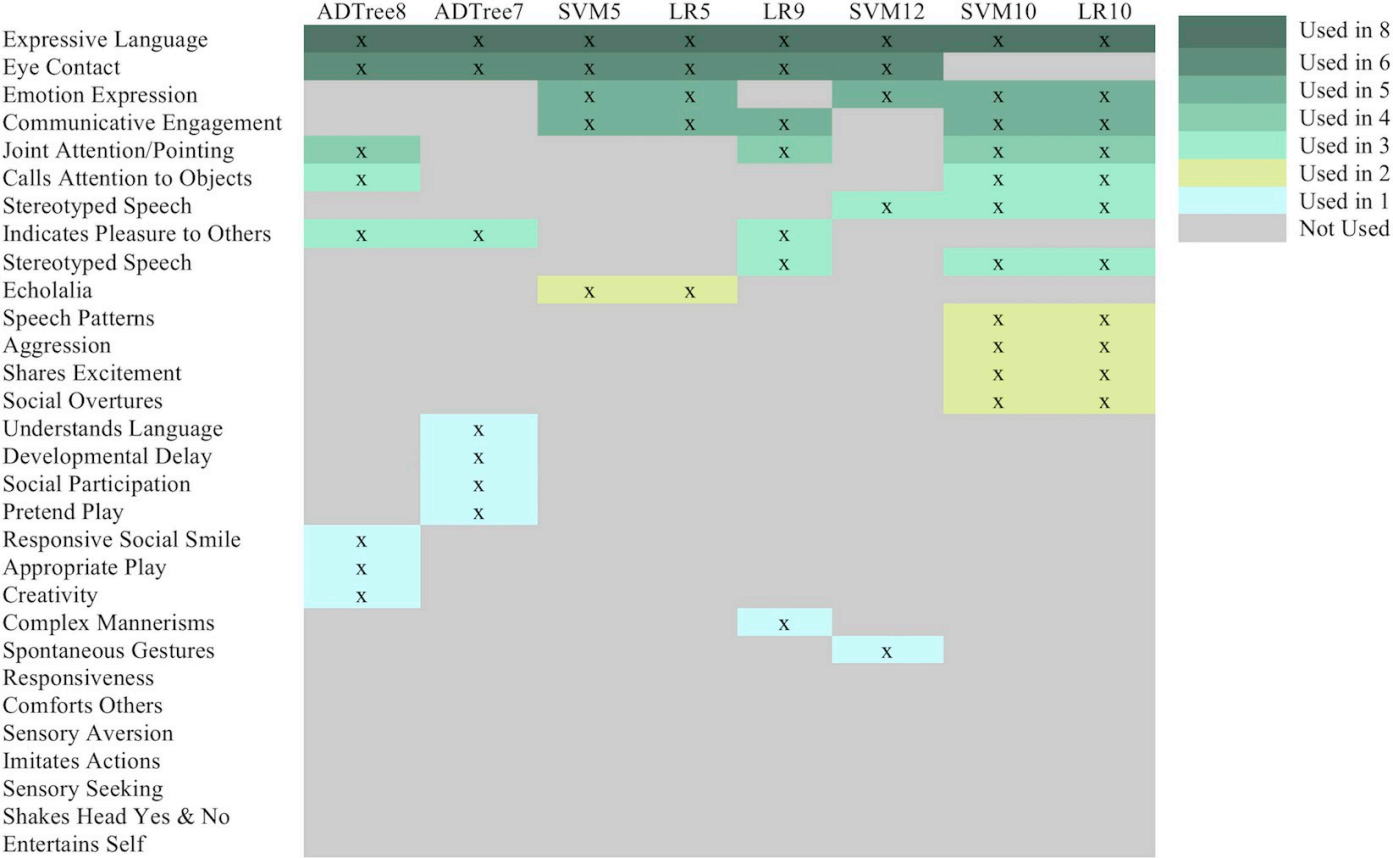
Feature-to-classifier mapping. Video analysts scored each video with 30 features. This matrix shows which feature corresponds to which classifier. Darker colored features indicate higher overlap, and lighter colors indicate lower overlap across the models. The features are rank ordered according to their frequency of use across the 8 classifiers. Further details about the classifiers are provided in Table 1. The bottom 7 features were not part of the machine learning process but were chosen because of their potential relationship with the autism phenotype and for use in further evaluation of the models’ feature sets when constructing a video feature–specific classifier. ADTree7, 7-feature alternating decision tree; ADTree8, 8-feature alternating decision tree; LR5, 5-feature logistic regression classifier; LR10, 10-feature logistic regression classifier; SVM5, 5-feature support vector machine; SVM10, 10-feature support vector machine; SVM12, 12-feature support vector machine.

Accounting for overlap in the features selected, these 8 models measure 23 unique features in total. The test accuracy for each model was >90%. All models contain approximately 90% fewer questions than the ADI-R and 70%–84% fewer questions than the total features measured within the ADOS. An additional 7 features were chosen for their potential diagnostic value and scored by video raters to assess their suitability for scoring home videos, creating a total of 30 features for the mobile video rating process described below (Fig 1).

### Recruitment and video collection

Under an approved Stanford University IRB protocol, we developed a mobile portal to facilitate the collection of videos of children with ASD, from which participants electronically consented to participate and upload their videos. Participants were recruited via crowdsourcing methods [38–41] targeted at social media platforms and listservs for families of children with autism. Interested participants were directed to a secure and encrypted video portal website to consent to participate. We required participants to be at least 18 years of age and the primary care provider(s) for a child with autism between the ages of 12 months and 17 years. Participants provided videos either through direct upload to the portal or via reference to a video already uploaded to YouTube together with age, diagnosis, and other salient characteristics. We considered videos eligible if they (1) were between 1 and 5 minutes in length, (2) showed the face and hands of the child, (3) showed clear opportunities for or direct social engagement, and (4) involved opportunities for the use of an object such as a utensil, crayon, or toy.

We relied on self-reported information provided by the parents concerning the child’s official diagnosis of autism or non-autism, the age of the child when the video was submitted, and additional demographic information for videos that were submitted directly to the web portal. For videos that were provided via YouTube URLs, we used YouTube metatags to confirm the age and diagnosis of the child in the video. If a video did not include a metatag for the age of the child in the video, the age was assigned following full agreement among the estimates made by 3 clinical practitioners in pediatrics. To evaluate the accuracy of the parents’ self-report and to safeguard against reporting biases, we commissioned a practicing pediatric specialist certified to administer the ADOS to review a random selection of 20 videos. We also commissioned a developmental pediatrician to review a nonoverlapping random selection of 10 additional videos. These clinical experts classified each video as “ASD” or “non-ASD.”

### Feature tagging of videos to run machine learning models

We employed a total of 9 video raters who were either students (high school, undergraduate, or graduate-level) or working professionals. None had training or certification for detection or diagnosis of autism. All were given instructions on how to tag the 30 questions and were asked to score 10 example videos before performing independent feature tagging of new videos. After training, we provided the raters with unique usernames and passwords to access the secure online portal to watch videos and answer 30 questions for each video needed by the feature vectors to run the 8 machine learning classifiers (Table 1). Features were presented to the video raters as multiple-choice questions written at an approximately seventh-grade reading level. The raters, who remained blind to diagnosis throughout the study, were tasked to choose one of the tags for each feature that best described the child’s behavior in the video. Each response to a feature was then mapped to a score between 0 and 3, with higher scores indicating more severe autism features in the measured behavior, or 8 to indicate that the feature could not be scored. The behavioral features and the overlap across the models are provided in Fig 1.

**Table 1:** Eight machine learning classifiers used for video analysis and autism detection. The models were constructed from an analysis of archived medical records from the use of standard instruments, including the ADOS and the ADI-R. All 8 models identified a small, stable subset of features in cross-validation experiments. The total numbers of affected and unaffected control participants for training and testing are provided together with measures of accuracy on the test set. Four models were tested on independent datasets and have been mentioned in a separate “Test” category. The remaining 4, indicated with “Train/test,” used the given dataset with an 80%:20% train:test split to calculate test accuracy on the 20% held-out test set. The naming convention of the classifiers is “model type”-“number of features”.

Classifier	Medical record source	# features	*N*_ASD_	*N*_non-ASD_	Mean age (SD)	% Male (*N*)	Test sensitivity	Test specificity	Test accuracy
ADTree8 [30]	ADOS Module 1	8	Train: 612	Train:15	6.16 (4.16)	76.8% (*N* = 2,009)	100%	100%	100%
			Test [30]: 446	Test [30]: 0					
			Test [25]: 2,333	Test [25]: 238					
			Test [32]: 931	Test [32]: 102					
ADTree7 [29]	ADI-R	7	Train: 891	Train: 75	8.5 (3.3)	65% (*N* = 628)	100%	1.13%	99.9%
			Test [24]: 222	Test [24]: 0					
			Test [32]: 462	Test [32]: 218					
SVM with L1 norm (SVM5) [27]	ADOS Module 2	5	Train/test: 1,319	Train/test: 70	6.92 (2.83)	80% (*N* = 1,101)	98%	58%	98%
LR with L2 norm (LR5) [27]	ADOS Module 2	5	Train/test: 1,319	Train/test: 70	6.92 (2.83)	80% (*N* = 1,101)	93%	67%	95%
LR with L1 norm (LR9) [26]	ADOS Module 2	9	Train: 362	Train: 282	11.75 (10)	76.4% (*N* = 1,375)	98.81%	89.39%	98.27%
			Test: 1,089	Test: 66					
Radial kernel SVM (SVM12) [26]	ADOS Module 3	12	Train: 510	Train: 93	16.25 (11.58)	76.4% (*N* = 2,094)	97.71%	97.2%	97.66%
			Test: 1,924	Test: 214					
Linear SVM (SVM10) [27]	ADOS Module 3	10	Train/test: 2,870	Train/test: 273	9.08 (3.08)	81% (*N* = 2,557)	95%	87%	97%
LR (LR10) [27]	ADOS Module 3	10	Train/test: 2,870	Train/test: 273	9.08 (3.08)	81% (*N* = 2,557)	90%	89%	94%

Abbreviations: ADI-R, Autism Diagnostic Interview-Revised; ADOS, Autism Diagnostic Observation Schedule; ADTree7, 7-feature alternating decision tree; ADTree8, 8-feature alternating decision tree; LR, logistic regression; LR5, 5-feature LR classifier; LR10, 10-feature LR classifier; SVM, support vector machine; SVM5, 5-feature SVM; SVM10, 10-feature SVM; SVM12, 12-feature SVM.

To test the viability of feature tagging videos for rapid machine learning detection and diagnosis of autism, we empirically identified a minimum number of video raters needed to score parent-provided home videos. We selected a random subset of videos from the full set of videos collected through our crowdsourced portal and ran the ADTree8 [30] model on feature vectors tagged by all 9 raters. We chose to run only ADTree8 for efficiency reasons and because this model has been previously validated in 2 independent studies [25,32]. We used a sample-with-replacement permutation procedure to measure accuracy as a function of majority rater agreement with the true diagnostic classification. We incrementally increased the number of video raters per trial by 1 rater, starting with 1 and ending with 9, drawing with replacement 1,000 times per trial. When considering only 2 raters, we required perfect class agreement between the raters. With an odd number of raters, we required a strict majority consensus. When an even number of raters disagreed on classification, we used an independent and randomly chosen rater’s score to break the tie.

After determining the minimally viable number of video raters, we used that minimum to generate the full set of 30-feature vectors on all videos. Seven of the models were written in Python 3 using the package scikit-learn, and one was written in R. We ran these 8 models on our feature matrices after feature tagging on videos. We measured the model accuracy through comparison of the raters’ majority classification result with the true diagnosis. We evaluated model performance further by age categories: ≤2 years, >2 to ≤4 years, >4 years to ≤6 years, and >6 years. For each category, we calculated accuracy, sensitivity, and specificity.

We collected timed data from each rater for each video, which began when a video rater pressed “play” on the video and concluded when a video rater finished scoring by clicking “submit” on the video portal. We used these time stamps to calculate the time spent annotating each video. We approximated the time taken to answer the questions by excluding the length of the video from the total time spent to score a video.

### Building a video feature classifier

The process of video feature tagging provides an opportunity to generate a crowdsourced collection of independent feature measurements that are specific to the video of the child as well as independent rater impressions of that child’s behaviors. This in turn has the ability to generate a valuable feature matrix to develop models that include video-specific features rather than features identified through analysis on archived data generated through administration of the SOC (as is the case for all classifiers contained in Table 1). To this end, and following the completion of the annotation on all videos by the minimum number of raters, we performed machine learning on our video feature set. We used LR with an elastic net penalty [42] (LR-EN-VF) to predict the autism class from the non-autism class. We randomly split the dataset into training and testing, reserving 20% for the latter while using cross-validation on the training set to tune for hyperparameters. We used cross-validation for model hyperparameter tuning by performing a grid search with different values of alpha (varying penalty weights) and L1 ratio (the mixing parameter determining how much weight to apply to L1 versus L2 penalties). Based on the resulting area under the curve (AUC) and accuracy from each combination, we selected the top-performing pair of hyperparameters. Using this pair, we trained the model using LR and balanced class weights to adjust weights inversely proportional to class frequencies in the input data. After determining the top-ranked features based on the trained model and the resulting coefficients, we validated the model on the reserved test set.

### Independent test set for validation of video phenotyping processes

We used our video portal and crowdsourcing approaches to generate an independent collection of videos for evaluation and feature tagging by 3 different raters than those used in the primary analysis. These raters had similar characteristics to the original group (age, education, no clinical certifications in developmental pediatrics) and were trained for video tagging through the same procedures.

### Ethics statement

This study was conducted under approval by Stanford University’s IRB under protocol IRB-31099. Informed and written consent was obtained from all study participants who submitted videos to the study.

## Results

All classifiers used for testing the time and accuracy of mobile video rating had accuracies above 90% (Table 1). The union of features across these 8 classifiers (Table 1) was 23 (Fig 1). These features plus an additional 7 chosen for clinical validity testing were loaded into a mobile video rating portal to enable remote feature tagging by nonclinical video raters.

We collected a total of 193 videos (Table 2) with average video length of 2 minutes 13 seconds (SD = 1 minute 40 seconds). Of the 119 ASD videos, 72 were direct submissions made by the primary caregiver of the child, and 47 were links to an existing video on YouTube. Of the 74 non-ASD videos, 46 non-ASD videos were links to existing YouTube videos, and 28 were direct submissions from the primary caregiver. We excluded 31 videos because of insufficient evidence for the diagnosis (*n* = 25) or inadequate video quality (*n* = 6), leaving 162 videos (116 with ASD and 46 non-ASD) which were loaded into our mobile video rating portal for the primary analysis. To validate self-reporting of the presence or absence of an ASD diagnosis, 2 clinical staff trained and certified in autism diagnosis evaluated a random selection of 30 videos (15 with ASD and 15 non-ASD) from the 162 videos. Their classifications had perfect correspondence with the diagnoses provided through self-report by the primary caregiver.

**Table 2:** Demographic information on children in the collected home videos. We collected *N* = 193 (119 ASD, 74 non-ASD) home videos for analysis. We excluded 31 videos because of inadequate labeling or video quality. We used a randomly chosen 25 autism and 25 non-autism videos to empirically define an optimal number of raters. Video feature tagging for machine learning was then done on 162 home videos.

Videos	*N*	*N*_ASD_	*N*_non-ASD_	Mean age (SD)	≤2 years	>2 years and ≤4 years	>4 years and ≤6 years	>6 years	Percent male, ASD	Percent male, non-ASD
Total	193	119	74	4 years 4 months (2 years 1 month)	24.4% (*n* = 47)	33.7% (*n* = 65)	25.9% (*n* = 50)	15.5% (*n* = 31)	39.38% (*n* = 76)	23.32% (*n* = 45)
Excluded	31	3	28	3 years 8 months (1 year 11 months)	32.3% (*n* = 10)	29.0% (*n* = 9)	25.8% (*n* = 8)	9.6% (*n* = 4)	3.23% (*n* = 1)	58.06% (*n* = 18)
Total videos used for analysis of all 8 classifiers	162	116	46	4 years 4 months (2 years 2 months)	22.8% (*n* = 37)	34.5% (*n* = 56)	25.9% (*n* = 42)	16.7% (*n* = 27)	67.2% (*n* = 78)	56.5% (*n* = 26)
Subset of videos used to find minimally viable number of raters	50	25	25	4 years 6 months (2 years 4 months)	28% (*n* = 14)	34% (*n* = 17)	18% (*n* = 9)	20% (*n* = 10)	48% (*n* = 12)	44% (*n* = 11)

Abbreviation: ASD, autism spectrum disorder.

**Fig 2:**
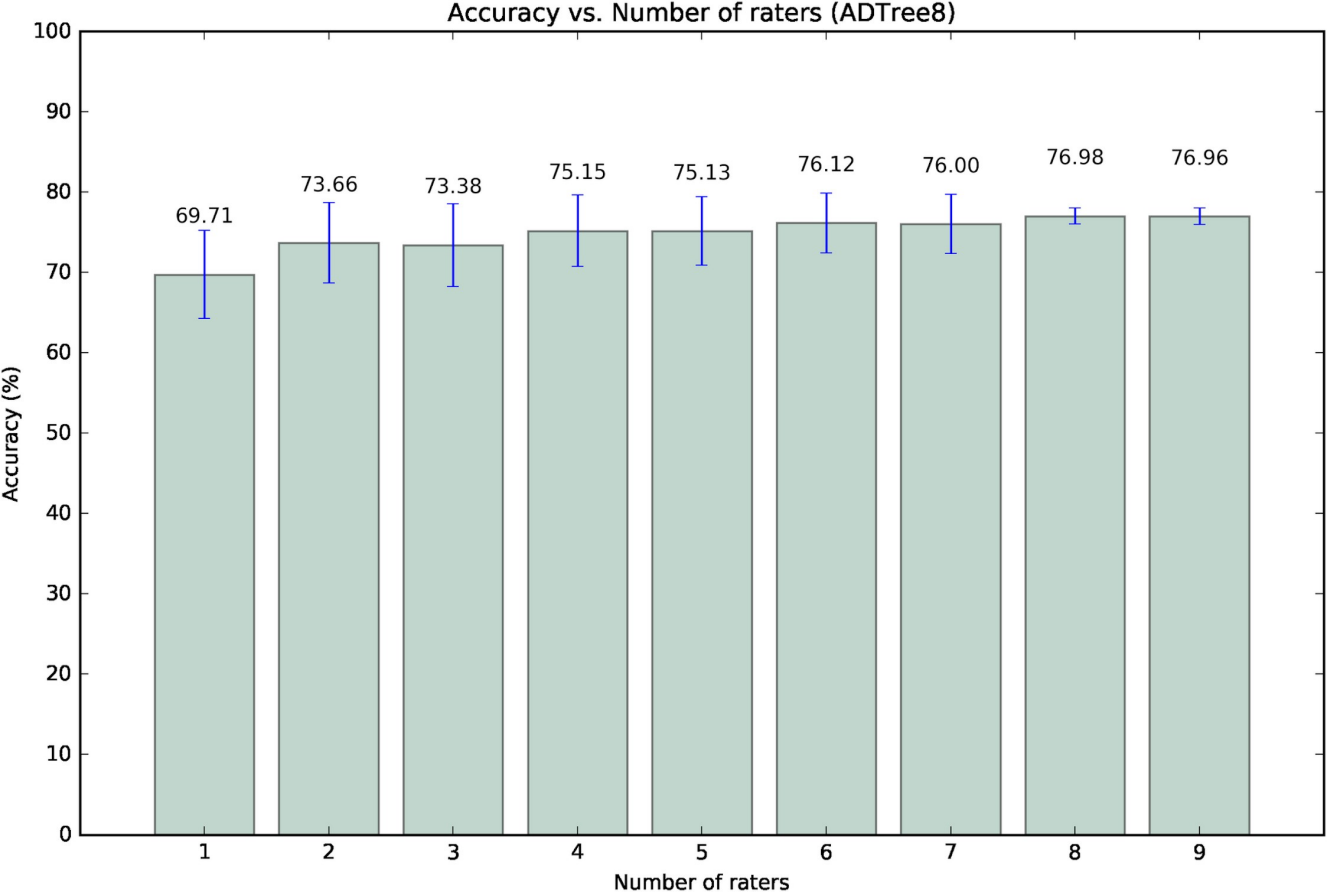
Accuracy across different permutations of 9 raters for 50 videos. We performed the analysis to determine the optimal number (the minimum number to reach a consensus on classification) of video raters needed to maintain accuracy without loss of power. Nine raters analyzed and generated feature tags for a subset of *n* = 50 videos (*n* = 25 ASD, *n* = 25 non-ASD) on which we ran the ADTree8 classifier (Table 1). The increase in accuracy conferred by the use of 3 versus 9 raters was not significant. We therefore set the optimal rater number to 3 for subsequent analyses. ADTree8, 8-feature alternating decision tree; ASD, autism spectrum disorder.

We randomly selected 50 videos (25 ASD and 25 non-ASD) from the total 162 collected videos and had 9 raters feature tag all in an effort to evaluate the potential for an optimal number of raters, with optimal being defined through a balance of scalability and information content. The average video length of this random subset was 1 minute 54 seconds (SD = 46 seconds) for the ASD class and 2 minutes 36 seconds (SD = 1 minute 15 seconds) for the non-ASD class. We then ran the ADTree8 (Table 1) model on the feature vectors generated by the 9 raters. We found the difference in accuracy to be statistically insignificant between 3 raters—the minimum number to have a majority consensus on the classification with no ties—and 9 raters (Fig 2). We therefore elected to use a random selection of 3 raters from the 9 to feature tag all 162 crowdsourced home videos.

### Model performance

**Fig 3:**
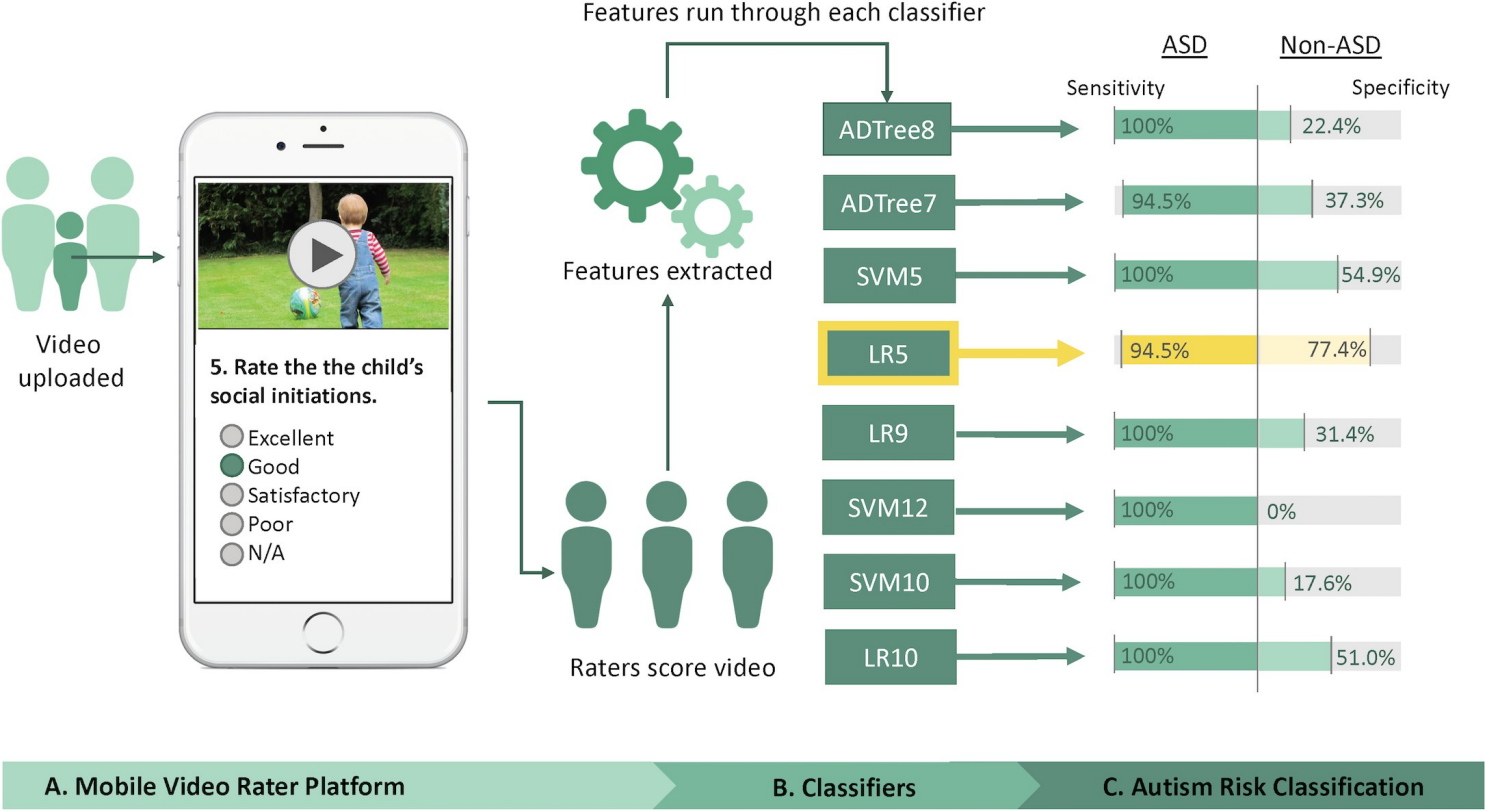
Overall procedure for rapid and mobile classification of ASD versus non-ASD and performance of models from Table 1. Participants were recruited to participate via crowdsourcing methods and provided video by direct upload or via a preexisting YouTube link. The minimum for majority rules of 3 video raters tagged all features, generating feature vectors to run each of the 8 classifiers automatically. The sensitivity and specificity based on majority outcome generated by the 3 raters on 162 (119 with autism) videos are provided. Highlighted in yellow is the best performing model, LR5. ADTree7, 7-feature alternating decision tree; ADTree8, 8-feature alternating decision tree; ASD, autism spectrum disorder; LR5, 5-feature logistic regression classifier; LR9, 9-feature logistic regression classifier; LR10, 10-feature logistic regression classifier; SVM5, 5-feature support vector machine; SVM10, 10-feature support vector machine; SVM12, 12-feature support vector machine.

Three raters performed video screening and feature tagging to generate vectors for each of the 8 machine learning models for comparative evaluation of performance (Fig 3). All classifiers had sensitivity >94.5%. However, only 3 of the 8 models exhibited specificity above 50%. The top-performing classifier was LR5, which showed an accuracy of 88.9%, sensitivity of 94.5%, and specificity of 77.4%. The next-best-performing models were SVM5 with 85.4% accuracy (54.9% specificity) and LR10 with 84.8% accuracy (51% specificity).

**Fig 4:**
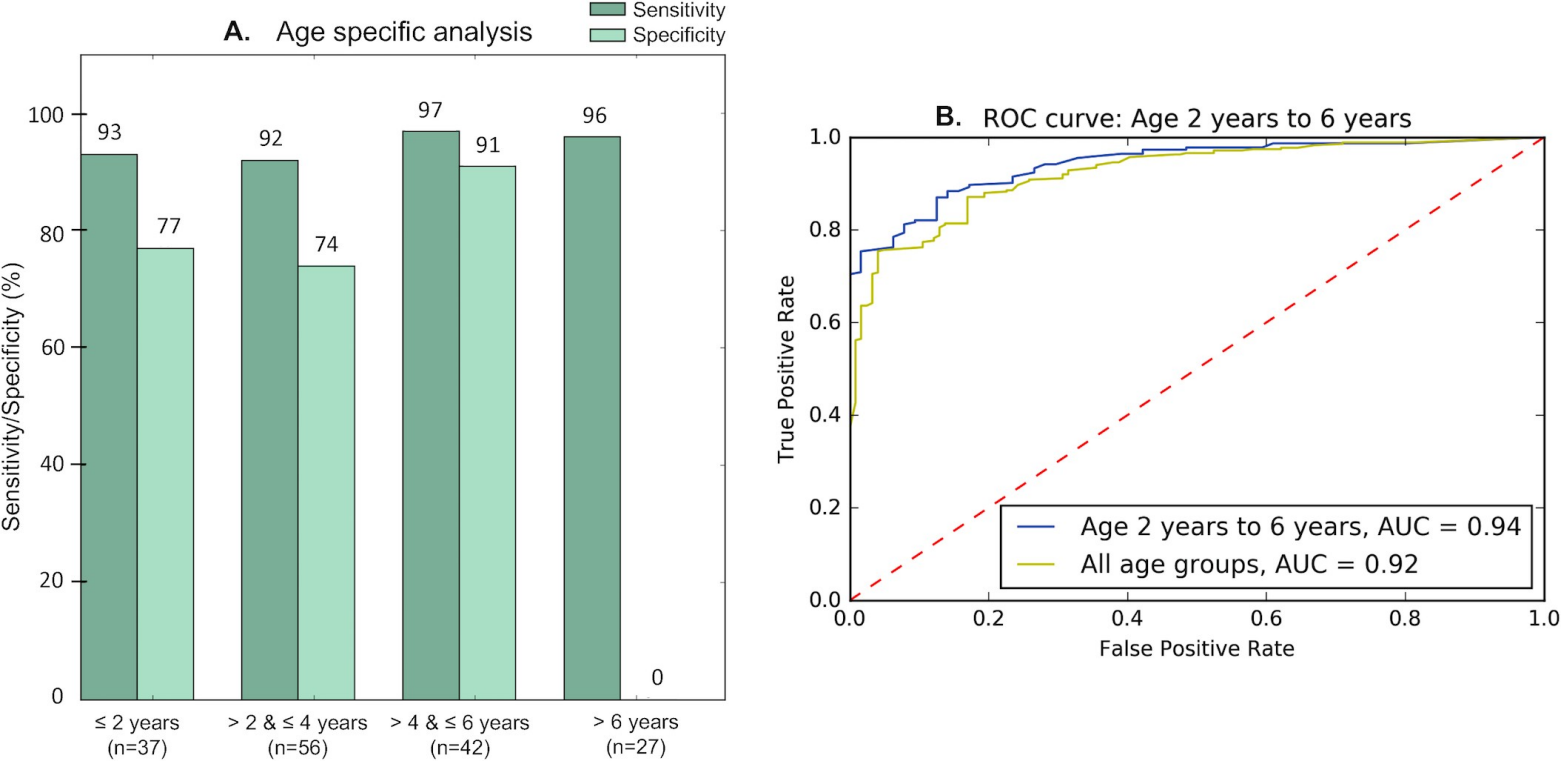
Performance for LR5 by age. LR5 exhibited the highest classifier performance (89% accuracy) out of the 8 classifiers tested (Table 1). This model performed best on children between the ages of 2 and 6 years. (A) shows the performance of LR5 across 4 age ranges, and (B) provides the ROC curve for LR5’s performance for children ages 2 to 6 years. Table 3 provides additional details, including the number of affected and unaffected control participants within each age range. AUC, area under the curve; LR5, 5-feature logistic regression classifier; ROC, receiver operating characteristic.

LR5 exhibited high accuracy on all age ranges with the exception of children over 6 years old (although note that we had limited examples of non-ASD [*n* = 1] class in this range). This model performed best on children between the ages of 4 and 6 years, with sensitivity and specificity both above 90% (Fig 4, Table 3). SVM5 and LR10 showed an increase in performance on children ages 2–4 years, both with 100% sensitivity and the former with 66.7% and the latter with 58.8% specificity. The 3 raters agreed unanimously on 116 out of 162 videos (72%) when using the top-performing classifier, LR5. The interrater agreement (IRA) for this model was above 75% in all age ranges with the exception of the youngest age group of children, those under 2 years, for which there was a greater frequency of disagreement. The numbers of non-ASD representatives were small for the older age ranges evaluated (Table 3).

**Table 3:** Model performance by age. This table details the accuracy, sensitivity, specificity, precision, and recall for 8 classifiers (Table 1) and for 4 age ranges found in evaluation of 162 home videos with an average length of 2 minutes. We also provide the IRA, which indicates the frequency with which the model results from all 3 raters’ feature tags agreed on class. The top-performing classifier was LR5, which yielded an accuracy of 88.9%, sensitivity of 94.5%, and specificity of 77.4%. Other notable classifiers were SVM5 and LR10, which yielded 85.4% and 84.8% accuracy, respectively. These 3 best-performing classifiers showed improved classification power within certain age ranges.

Age group	Statistic	ADTree8	ADTree7	SVM5	LR5	LR9	SVM12	SVM10	LR10
Overall (116 ASD, 46 non-ASD, 64.4% male)	Sensitivity	100%	94.5%	100%	94.5%	100%	100%	100%	100%
	Specificity	22.4%	37.3%	54.9%	77.4%	31.4%	0%	17.6%	51.0%
	Accuracy	76.1%	76.3%	85.4%	88.9%	78.4%	71.6%	73.9%	84.8%
	IRA	76.1%	67.5%	68.4%	71.7%	75.9%	71.6%	79.5%	70.9%
	Precision	74.3%	76.3%	82.3%	89.7%	76.0%	71.6%	72.4%	82.0%
	UAR	61.2%	65.9%	77.5%	86.0%	65.7%	50%	58.8%	75.5%
≤2 years (17 ASD, 20 non-ASD, 56.8% male)	Sensitivity	100%	100%	100%	93.3%	100%	100%	100%	100%
	Specificity	14.2%	18.2%	38.1%	77.3%	22.7%	0%	14.3%	47.6%
	Accuracy	50%	51.4%	62.9%	83.8%	54.1%	40.5%	50.0%	96.2%
	IRA	53%	48.6%	45.7%	51.4%	48.6%	100%	66.7%	100%
	Precision	45.5%	45.5%	51.9%	73.7%	46.9%	45.9%	45.4%	57.7%
	UAR	57.1%	59.1%	69.1%	85.3%	61.4%	50.0%	57.2%	73.8%
>2 years and ≤4 years (39 ASD, 17 non-ASD, 66.1% male)	Sensitivity	100%	97.3%	100%	91.8%	100.0%	100%	100%	100%
	Specificity	23.6%	50%	66.7%	73.7%	38.9%	0%	22.2%	58.8%
	Accuracy	76.4%	50%	88.9%	85.7%	80.4%	66.7%	74.5%	86.8%
	IRA	74.5%	81.8%	63.0%	75.0%	76.8%	100%	85.4%	77.4%
	Precision	74.5%	80.0%	85.7%	87.2%	77.6%	69.6%	72.5%	83.7%
	UAR	61.8%	73.7%	83.4%	82.8%	69.5%	50.0%	61.1%	79.4%
>4 years and ≤6 years (34 ASD, 8 non-ASD, 61.9% male)	Sensitivity	100%	96.8%	100%	96.9%	100.0%	100%	100%	100%
	Specificity	40.0%	60%	72.7%	90.9%	40.0%	0%	18.2%	50.0%
	Accuracy	85.4%	87.8%	92.9%	95.3%	85.7%	74.4%	79.1%	88.1%
	IRA	85.4%	78.0%	78.6%	79.1%	85.7%	93.0%	76.7%	71.4%
	Precision	83.8%	88.2%	91.2%	96.9%	84.2%	80.9%	78.0%	86.5%
	UAR	70.0%	78.4%	86.4%	93.9%	70.0%	50.0%	59.1%	75.0%
>6 years (26 ASD, 1 non-ASD, 74.1% male)	Sensitivity	100%	84.6%	100%	96.2%	100%	100%	100%	100%
	Specificity	0%	0%	0%	0%	0%	0%	0%	0%
	Accuracy	96.2%	81.5%	96.2%	92.6%	96.2%	96.2%	96.2%	96.3%
	IRA	96.2%	70.4%	96.2%	81.5%	96.2%	100%	100%	85.2%
	Precision	96.3%	95.7%	96.3%	96.2%	96.3%	96.3%	96.3%	96.3%
	UAR	50.0%	42.3%	50.0%	48.1%	50.0%	50.0%	50.0%	50.0%

Abbreviations: ADTree7, 7-feature alternating decision tree; ADTree8, 8-feature alternating decision tree; ASD, autism spectrum disorder; IRA, interrater agreement; LR5, 5-feature logistic regression classifier; LR9, 9-feature logistic regression classifier; LR10, 10-feature logistic regression classifier; SVM5, 5-feature support vector machine; SVM10, 10-feature support vector machine; SVM12, 12-feature support vector machine; UAR, unweighted average recall.

The median time for the 3 raters to watch and score a video was 4 minutes (Table 4). Excluding the time spent watching the video, raters required a median of 2 minutes 16 seconds to tag all 30 features in the analyst portal. We found a significant difference (*p* = 0.0009) between the average time spent to score the videos of children with ASD and the average time spent to score the non-ASD videos (6 minutes 36 seconds compared with 5 minutes 8 seconds).

**Table 4:** Time required for mobile tagging of video features needed to run the machine learning models. We highlight the average length of videos (all participants, only participants with ASD, and only participants without ASD) as well as the average time required to watch and score the videos and the average time required from start to end of the scoring component alone.

		Total time required for review and feature tagging	Total time required for feature tagging alone	Video length
Overall	Mean (SD)	6 minutes 9 seconds (5 minutes 28 seconds)	3 minutes 36 seconds (5 minutes 52 seconds)	2 minutes 13 seconds (1 minute 40 seconds)
	Median	4 minutes 0 seconds	2 minutes 16 seconds	1 minute 45 seconds
	Range	1 minute 0 seconds to 37 minutes 0 seconds	0 minutes 50 seconds to 35 minutes 42 seconds	0 minutes 25 seconds to 8 minutes 6 seconds
ASD only	Mean (SD)	6 minutes 36 seconds (5 minutes 54 seconds)	4 minutes 22 seconds (6 minutes 20 seconds)	2 minutes 4 seconds (1 minute 40 seconds)
	Median	5 minutes 0 seconds	2 minutes 40 seconds	1 minute 30 seconds
	Range	1 minute 0 seconds to 37 minutes 0 seconds	0 minutes 50 seconds to 35 minutes 42 seconds	0 minutes 25 seconds to 8 minutes 6 seconds
Non-ASD only	Mean (SD)	5 minutes 8 seconds (4 minutes 8 seconds)	2 minutes 18 seconds (4 minutes 22 seconds)	2 minutes 38 seconds (1 minute 34 seconds)
	Median	4 minutes 0 seconds	1 minute 21 seconds	2 minutes 11 seconds
	Range	1 minute 0 seconds to 30 minutes 0 seconds	0 minutes 50 seconds to 25 minutes 42 seconds	0 minutes 36 seconds to 6 minutes 42 seconds

Abbreviation: ASD, autism spectrum disorder.

### Independent validation

To validate the feasibility and accuracy of rapid feature tagging and machine learning on short home videos, we launched a second effort for crowdsourcing videos of children with and without autism to generate an independent replication dataset. We collected 66 videos, 33 of children with autism and 33 non-ASD. This set of videos was comparable to the initial set of 162 videos in terms of gender, age, and video length. The average age for children with ASD was 4 years 5 months (SD = 1 year 9 months), and the average age for non-ASD children was 3 years 11 months (SD = 1 year 7 months). Forty-two percent (*n* = 14) of the children with ASD were male and 45% (*n* = 15) of the non-ASD children were male. The average video length was 3 minutes 24 seconds, with an SD of 45 seconds. For this independent replication, we used 3 different raters, each with no official training or experience with developmental pediatrics. The raters required a median time of 6 minutes 48 seconds for complete feature tagging. LR5 again yielded the highest accuracy, with a sensitivity of 87.8% and a specificity of 72.7%. A total of 13 of the 66 videos were misclassified, with 4 false negatives.

Given the higher average time for video evaluation, we hypothesized that the videos contained challenging displays of autism symptoms. Therefore, we examined the probabilities generated by the LR5 model for the 13 misclassified videos. Two of the 4 false negatives and 4 of the 9 false positives had borderline probabilities scores between 0.4 and 0.6. We elected to define a probability threshold between 0.4 and 0.6 to flag videos as inconclusive cases. Twenty-six of the 66 videos fell within this inconclusive group when applying this threshold. When we excluded these 26 from our accuracy analysis, the sensitivity and specificity increased to 91.3% and 88.2%, respectively.

### Training a video feature–specific classifier

**Fig 5:**
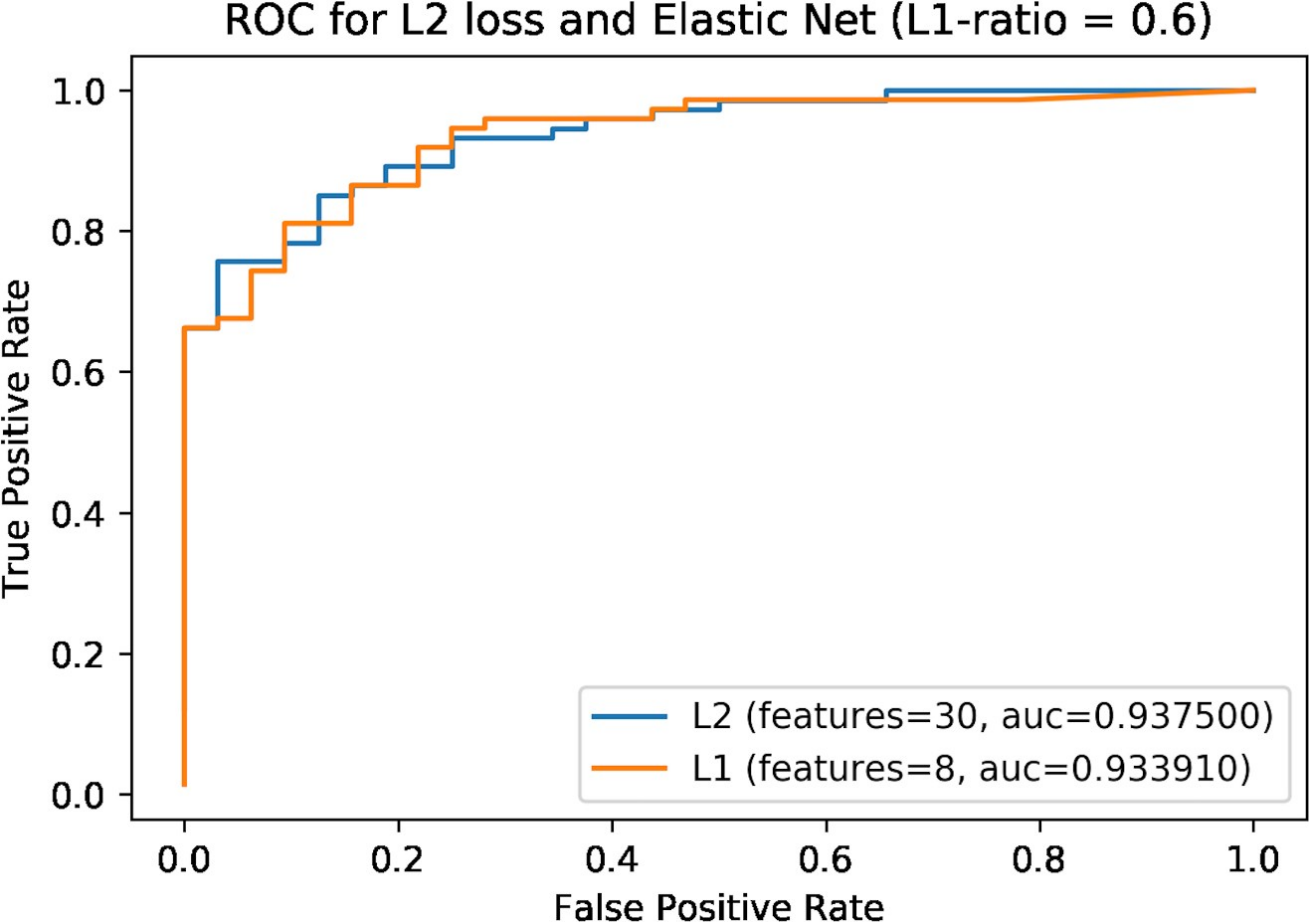
ROC curve for LR-EN-VF showing performance on test data along with an ROC for L2 loss with no feature reduction. The former chose 8 out of 30 video features. AUC, area under the curve; LR-EN-VF, logistic regression with an elastic net penalty; ROC, receiver operating characteristic.

To build a video feature–specific classifier, we trained an LR-EN-VF model on 528 (3 raters × 176 videos) novel measures of the 30 video features used to distinguish the autism class from the neurotypical cohort. Out of these 176 videos (ASD = 121, non-ASD = 58), 162 (ASD = 116, non-ASD = 46) were from the analysis set, and 14 videos (ASD = 5, non-ASD = 12) were from the set of 66 validation videos. Model hyperparameters (alpha and L1 ratio) identified through 10-fold cross-validation were 0.01 and 0.6, respectively. We used a high L1 ratio to enforce sparsity and to decrease model complexity and the number of features. We had similar proportions (0.60) for non-ASD and ASD measures in the training set and held-out test set, which allowed us to create a model that generalizes well without a significant change in sensitivity or specificity on novel data. The model had an area under the receiver operating characteristic curve (AUC-ROC) of 93.3% and accuracy of 87.7% on the held-out test set. A comparison of LR-EN-VF with LR L2 penalty (no feature reduction) revealed similar results (AUC-ROC: 93.8%, test accuracy: 90.7%) (Fig 5). The top-8 features selected by the model consisted of the following, in order of highest to lowest rank: speech patterns, communicative engagement, understands language, emotion expression, sensory seeking, responsive social smile, stereotyped speech. One of these 8 features—sensory seeking—was not part of the full sets of items on the standard instrument data used in the development and testing of the 8 models depicted in Table 1. We then validated this classifier on the remaining 52 videos (ASD = 28, non-ASD = 21) from the validation set, and the results showed an accuracy of 75.5% and an AUC-ROC of 86.0%.

## Discussion

Previous work [26–29] has shown that machine learning models built on records from standard autism diagnoses can achieve high classification accuracy with a small number of features. Although promising in terms of their minimal feature requirements and ability to generate an accurate risk score, their potential for improving autism diagnosis in practice has remained an open question. The present study tested the ability to reduce these models to the practice of home video evaluation by nonexperts using mobile platforms (e.g., tablets, smartphones). Independent tagging of 30 features by 3 raters blind to diagnosis enabled majority rules machine learning classification of 162 two-minute (average) home videos in a median of 4 minutes at 90% AUC on children ages 20 months to 6 years. This performance was maintained at 89% AUC (95% CI 81%–95%) in a prospectively collected and independent external set of 66 videos each with 3 independent rater measurement vectors. Taking advantage of the probability scores generated by the best-performing model (L1-regularized LR model with 5 features) to flag low-confidence cases, we were able to achieve a 91% AUC, suggesting that the approach could benefit from the use of the scores on a more quantitative scale rather than just as a binary classification outcome.

By using a mobile format that can be accessed online, we showed that it is possible to get multiple independent feature vectors for classification. This has the potential to elevate confidence in classification outcome at the time of diagnosis (i.e., when 3 or more agree on class) while fostering the growth of a novel matrix of features from short home videos. In the second part of our study, we tested the ability for this video feature matrix to enable development of a new model that can generalize to the task of video-based classification of autism. We found that an 8-feature LR model could achieve an AUC of 0.93 on the held-out subset and 0.86 on the prospective independent validation set. One of the features used by this model, sensory seeking, was not used by the instruments on which the original models were trained, suggesting the possibility that alternative features may provide added power for video classification.

These results support the hypothesis that the detection of autism can be done effectively at scale through mobile video analysis and machine learning classification to produce a quantified indicator of autism risk quickly. Such a process could streamline autism diagnosis to enable earlier detection and earlier access to therapy that has the highest impact during earlier windows of social development. Further, this approach could help to reduce the geographic and financial burdens associated with access to diagnostic resources and provide more equal opportunity to underserved populations, including those in developing countries. Further testing and refinement should be conducted to identify the most viable method(s) of crowdsourcing video acquisition and feature tagging. In addition, prospective trials in undiagnosed and in larger, more-balanced cohorts including examples of children with non-autism developmental delays will be needed to better understand the approach’s potential for use in autism diagnosis.

## Supporting information

S1 Table Results of 8 classifiers on independent validation set. LR10, LR5, and ADTree7 are the top-3 best-performing classifiers on the validation set, which falls in line with the results observed on the test dataset of 162 videos used earlier. LR5 still performs with the highest specificity out of the 8 models. ADTree7, 7-feature alternating decision tree; LR5, 5-feature logistic regression classifier; LR10, 10-feature logistic regression classifier.
(DOCX)

S1 Text Instructions for video raters. (DOCX)

S1 Checklist The tripod checklist. (DOCX)

## Abbreviations

ADI-R: Autism Diagnostic Interview-Revised
ADOS: Autism Diagnostic Observation Schedule
ADTree: alternating decision tree
ADTree7: 7-feature alternating decision tree
ADTree8: 8-feature alternating decision tree
ASD: autism spectrum disorder
AUC: area under the curve
AUC-ROC: area under the receiver operating characteristic curve
BID: Balanced Independent Dataset
IRA: interrater agreement
LR: logistic regression
LR10: 10-feature logistic regression classifier
LR5: 5-feature logistic regression classifier
LR9: 9-feature logistic regression classifier
LR-EN-VF: logistic regression with an elastic net penalty
ROC: receiver operating characteristic
SOC: standard of care
SVM: support vector machine
SVM10: 10-feature support vector machine
SVM12: 12-feature support vector machine
SVM5: 5-feature support vector machine
UAR: unweighted average recall
