# Supplementary material for: Mobile detection of autism through machine learning on home video: A development and prospective validation study
Source: PLoS Med. 2018 Nov 27;15(11):e1002705. doi: 10.1371/journal.pmed.1002705 (PMC6258501; doi:10.1371/journal.pmed.1002705)
Supplement: S1 Table — LR10, LR5, and ADTree7 are the top-3 best-performing classifiers on the validation set, which falls in line with the results observed on the test dataset of 162 videos used earlier. LR5 still performs with the highest specificity out of the 8 models. ADTree7, 7-feature alternating decision tree; LR5, 5-feature logistic regression classifier; LR10, 10-feature logistic regression classifier. (DOCX) [file pmed.1002705.s001.docx]

| **Classifier** | **Performance on Validation set** |
| --- | --- |
| ADTree8 | Sensitivity: 93.9%, Specificity: 52.9%, UAR = 73.4% |
| ADTree7 | Sensitivity: 93.9%, Specificity: 60.6%, UAR = 77.3% |
| SVM12 | Sensitivity: 100.0%, Specificity: 41.2%, UAR = 70.6% |
| LR9 | Sensitivity: 100.0%, Specificity: 38.2%, UAR = 69.1% |
| SVM5 | Sensitivity: 100.0%, Specificity: 0%, UAR = 50% |
| LR5 | Sensitivity: 87.8%, Specificity: 72.7%, UAR = 80.3% |
| SVM10 | Sensitivity: 100.0%, Specificity: 41.2%, UAR = 70.6% |
| LR10 | Sensitivity: 93.9%, Specificity: 70.6%, UAR = 82.3% |
