# Supplementary material for: Mobile detection of autism through machine learning on home video: A development and prospective validation study
Source: PLoS Med. 2018 Nov 27;15(11):e1002705. doi: 10.1371/journal.pmed.1002705 (PMC6258501; doi:10.1371/journal.pmed.1002705)
Supplement: S1 Text — (DOCX) [file pmed.1002705.s002.docx]

*Instructions for Video Raters:*

All video raters first completed HIPAA compliance and CITI training prior to scoring videos. Video raters reviewed all 30 questions with the training manager and discussed the features that were present in each question to clarify any misconceptions and to define any unknown terms (i.e. “echolalia,” what is meant by “spontaneous,” etc.). All video raters then watched two youtube videos alongside the training manager (the first video was of a 2-year-old neurotypical girl, and the second video was of a 2-year old girl with autism), and subsequently went through each of the 30 questions together as they apply to each video, again clarifying any misconceptions. Then, video raters were asked to score a standard sample of 10 youtube videos independently and review any more questions with the training manager.
