# Supplementary material for: Mobile detection of autism through machine learning on home video: A development and prospective validation study
Source: PLoS Med. 2018 Nov 27;15(11):e1002705. doi: 10.1371/journal.pmed.1002705 (PMC6258501; doi:10.1371/journal.pmed.1002705)
Supplement: S1 Checklist — (DOCX) [file pmed.1002705.s003.docx]

| Section/Topic | Item | Section | Paragraph number |
| --- | --- | --- | --- |
| Title | 1 | Title | 1 |
| Abstract | 2 | Abstract | 1 |
| Introduction |  |  |  |
| Background and objectives | 3a | Introduction | 1-2 |
|  | 3b | Introduction | 3-7 |
| Methods |  |  |  |
| Source of Data | 4a | Methods  *Subsection:* Recruitment and Video Collection | 1 |
|  | 4b | N/A | N/A |
| Participants | 5a | Methods  *Subsection:* Recruitment and Video Collection | 2 |
|  | 5b | Methods  *Subsection:* Recruitment and Video Collection | 1 |
|  | 5c | Methods  *Subsection:* Recruitment and Video Collection | 2 |
| Outcome | 6a | Methods  *Subsection:*  Source Classifiers for Reduce to practice Testing | 1 |
|  | 6b | Methods  *Subsection:*  Source Classifiers for Reduce to practice Testing | 3-8 |
| Predictors | 7a | Methods  *Subsection:*  Feature tagging of videos to run machine learning models | 1 |
|  | 7b | Methods  *Subsection:*  Feature tagging of videos to run machine learning models | 1 |
| Sample size | 8 | Methods  *Subsection:*  Feature tagging of videos to run machine learning models | 1-3 |
| Missing Data | 9 | Methods  *Subsection:* Recruitment and Video Collection | 1-2 |
| Statistical analysis and methods | 10a | Methods  *Subsection:*   1. Source Classifiers for Reduce to practice Testing 2. Building a video feature classifier | 1. 3-8 2. 1 |
|  | 10b | Methods  *Subsection:*  Building a video feature classifier | Table 1 & para 1 of subsection |
|  | 10c | Methods  *Subsection:*  Independent test set for validation of video phenotyping processes | 1 |
|  | 10d | Methods | Table 1 |
|  | 10e | Methods  *Subsection:*  Building a video feature classifier | 1 |
| Risk groups | 11 | N/A | N/A |
| Development vs. Validation | 12 | Methods  *Subsection:*  Independent test set for validation of video phenotyping processes | 1 |
| Results |  |  |  |
| Participants | 13a | Results | 1-2 |
|  | 13b | Results | Table 2 |
|  | 13c | Results  *Subsection*: Independent Validation | 1 |
| Model development | 14a | Results | Table 3 |
|  | 14b | N/A | N/A |
| Model specification | 15a | Results  *Subsection:* Training a video-feature specific classifier | 1 |
|  | 15b | Results  *Subsection:* Training a video-feature specific classifier | 1 |
| Model performance | 16 | Results | Table 3, Fig 4 and Fig 5 |
| Model-updating | 17 | N/A | N/A |
| Discussion |  |  |  |
| Limitations | 18 | Discussion | 4 |
| Interpretations | 19a | Discussion | 2-3 |
|  | 19b | Discussion | 7 |
| Implications | 20 | Discussion | 6 |
| Other information |  |  |  |
| Supplementary information | 21 | Supplementary Materials | 1-2 |
| Funding | 22 | Entered in the Financial Disclosure section of the submission system. |  |
